# Why is public science education important?

**DOI:** 10.1186/1479-5876-4-7

**Published:** 2006-01-24

**Authors:** Elizabeth Marincola

**Affiliations:** 1 President, Science Service, Washington, D.C

## Background

For most Americans, science is something to be tolerated in high school, details of which are promptly forgotten after tests are over. This may be understandable, since, regrettably, the basic science curriculum can often consist of lectures on taxonomy or analogous facts about what science has discovered, along with the painful need to memorize long lists of strange words. But any notion that science should be left to the scientists, and that the very question of what is and is not science should be left to those with a political agenda, is wrong and damaging.

As the pace of scientific research accelerates, the average citizen is faced increasingly with having to grapple with matters of science in his everyday life. Some of the country's most complicated and urgent public policy debates have at their center been questions of science. It is imperative that the public is engaged in science issues which have an impact on their lives, in their own self-interest, to best thrive in modern society. Furthermore, citizens must understand what is a question *of *science, and what is a question of public policy that can be informed *by *science. For example, the many causes and effects that impact human health are questions of science: smoking is a cause of lung cancer; obesity is a cause of diabetes; lead poisoning is a cause of brain damage in the young; alcohol and drug use by pregnant women are a cause of brain damage to their unborn children. These are objectively proven claims and therefore are science. The public must also grapple with important public policy questions that must be informed *by *science. For example, an understanding of the science of embryonic stem cell research is critically important to inform policymakers who are advocating or opposing this research; an understanding of climatology is essential to those concerned with regulation of fossil fuel consumption and energy policy; astronomy and cosmology must inform wise investment in space exploration.

On a less weighty level, science is everywhere in society; a part of each person's everyday life – even grocery shopping is more informed by a basic understanding of science. But most citizens are not equipped to personally assess the facts, nor often even to separate the facts from opinion or political spin; science from non-science. They therefore are likely to be predominantly influenced on these issues by the prevailing perception in their communities.

Yet no country, no matter how sophisticated technologically, can advance its society fully without the informed engagement of its citizenship. The existence of a democratic process (voting rights, a transparent and representative governance structure) is necessary but not sufficient. As with economic decision-making, public policy decision-making depends on full information. The nonscientist is increasingly at a disadvantage because he lacks the information to engage in these important public policy dilemmas as an informed, independent thinker.

How can we equip our people with sufficient scientific skills to enable them to develop informed opinions about these important issues, without imposing the unrealistic expectation that they be trained as scientists? This question is distinct from the question of how the U.S. can continue to produce the world's leading scientists. The latter consideration is also of course critical to the future health and economic prosperity of the Nation. But without a broad populace of "science appreciators", both the continued national investment in science and the implementation of enlightened public policy will be threatened.

## Teach thinking more than facts

Distinguished biochemist Bruce Alberts, who served as President of the U.S. National Academy of Sciences from 1993 until 2005, highlights the importance of state science testing^1^. The "No Child Left Behind" Act mandates that effective in 2007, "high-stakes science assessments will be coming to all of our K-12 schools. It is left to each state to decide what science tests it will select for all its students [1]." History shows that, when pressured, science teachers adjust their curricula to ensure the best possible results on state tests [1]. Therefore it is imperative that scientists and policymakers get involved in the development of state science tests. This may be our last, best chance to influence how a generation recognizes science and what it understands about science. It will be counterproductive to squander this segment of their education on requiring extensive memorization of facts. Instead, state tests should teach students how to bring their own independent thinking to important issues. Thus, for example, most biology classes today stress the importance of having students learn names for the parts of an organism – with even seventh grade textbooks highlighting words like *endoplasmic reticulum, mitochondrion*, and *Golgi apparatus*. But it is much more important for students to experience the scientific method, so as to learn about the difference between data and speculation, how to frame a question, and how to approach a problem critically and skeptically. As called for in the National Science Education Standards of the U.S. National Academy of Sciences [2], this approach emphasizes logical, hands-on problem-solving, and insists on evidence for claims that can be confirmed by others. Had this requirement been broadly implemented in this country a generation ago, the painful and contentious debate over the teaching of creationism, "creation science" and "intelligent design" in public science class may have been unnecessary: at the core of this issue is the simple fact that these ideas, while they may or may not be true, do not present confirmable claims and therefore are not science. The public must be able to consider questions such as this within a framework that enables individuals to distinguish science from other propositions. Science education at all levels should focus on creating a society where well-educated adults are equipped to bring scientific thinking to bear on issues that affect them as citizens.

## Scientists must engage society

Scientists, writ large, can play a major role in the engagement of the public in science affecting their lives. We must resist the notion that a scientifically-trained person who does not do science *per se *for a living has "failed" as a scientist or even "abandoned" their science. Instead, we must urge "scientists" to become opinion leaders and policymakers. "Scientists" for these purposes include not just those with advanced training in a scientific discipline, but also the high school science fair student and the college biology major. When people who have experienced science become journalists, filmmakers and public servants, they bring rigor and scientific thinking to their work, and positively influence others to do the same.

Those who do dedicate their careers to science carry an even greater burden to engage their relatives, friends, neighbors and others in their communities. They must communicate why science is central to everyday life in terms that laypeople can understand, starting with why what they do is relevant. If a scientist cannot explain to a ten-year-old what he does and why it is relevant to the child, it's like a tree falling in the forest with no one there to hear it: it may happen, but nobody will care. Publicly-funded scientists must justify tax support, and privately-funded scientists must justify commercial investment. Furthermore, scientists who do not engage the public – by submitting op-eds to their local newspapers or calling into radio talk shows when timely issues arise; by volunteering to make a presentation in a local school, or by writing to or even meeting with their Member of Congress to discuss policy issues that are informed by science – in effect relinquish their expertise to non-experts: even our judicial system has increasingly and alarmingly been called upon to act as untrained and unqualified arbiters of science in questions of guilt and law [3].

## Increase the national investment in the public engagement in science

The Nation must invest heavily in engaging the public in science in parallel with our investments in the conduct of science itself. When people are left behind in their understanding of how public dollars are invested, their commitment to that funding is diminished. A disastrous recent example is the need to reinforce the levees protecting New Orleans. The Administration and Congress were able to quietly reduce the city's natural disaster preparedness budget through the Army Corps of Engineers [4] because there was insufficient public education about the need for this investment – and therefore insufficient resistance to reducing funding by the taxpaying public. Likewise, over the long run, the public funding of scientific research will depend on our investments in the public engagement in science. NASA may be the most successful government example of how public education about the importance of science has directly driven public funding to carry out its work. Its website [5] brings the agency's science to the desktops of all citizens, enabling them to appreciate the public investment in space exploration in real time.

We must consistently and clearly educate the public about what science is and is not, and how it benefits the citizenship. This responsibility is one that is spread among many industries and professions. For our future success as a nation, the media, professional scientists, industry, educators and many others must all become science communicators. The progression of basic to applied science to useful technologies, and, in medicine, from cellular to clinical research to useful disease treatments and preventions, depends on an informed public [6]. This is because ultimately it is the public that controls both the money and the policies that enable modern science and medicine to progress. That which a person does not understand, he tends to reject. We must engage the public in the challenges presented by science and medicine, to capture their imagination and hope, and to gain their essential support.

Elizabeth Marincola, President , Science Service, and Publisher Science News 1719 N Street, NW Washington, D.C. 20036 202-785-2255. emarincola@sciserv.org
